# Unfavorable Mealtime, Meal Skipping, and Shiftwork Are Associated with Circadian Syndrome in Adults Participating in NHANES 2005–2016

**DOI:** 10.3390/nu16111581

**Published:** 2024-05-23

**Authors:** Zoha Akbar, Zumin Shi

**Affiliations:** Human Nutrition Department, College of Health Sciences, QU Health, Qatar University, Doha P.O. Box 2713, Qatar; za1404491@qu.edu.qa

**Keywords:** diet, mealtime, meal skipping, shiftwork, chrononutrition, circadian rhythms, circadian syndrome, NHANES

## Abstract

The concept of Circadian Syndrome (CircS) aims to emphasize the circadian disruptions underlying cardiometabolic conditions. Meal timing and shiftwork may disrupt circadian rhythms, increasing cardiometabolic risk. This study aimed to assess the associations of meal timing, meal skipping, and shiftwork with CircS in US adults and explore effect modifications by sociodemographic and lifestyle factors. CircS was defined using Metabolic Syndrome components in addition to short sleep and depression symptoms. Data from 10,486 participants of the National Health and Nutrition Examination Survey 2005–2016 were analyzed cross-sectionally. Mealtime was assessed by calculating the midpoint of intake between breakfast and dinner and dichotomizing it into favorable mealtime (between 12:30 and 13:15) and unfavorable mealtime using a data-driven approach. Meal skippers were categorized separately. Participants working evening, night, or rotating shifts were classified as shift workers. In the multivariable logistic regression analysis, an unfavorable mealtime, meal skipping, and shiftwork were associated with a higher likelihood of CircS (OR = 1.24; 95%CI 1.07–1.44, OR = 1.39; 95%CI 1.16–1.67, and OR = 1.37; 95%CI 1.01–1.87, respectively). Subgroup analyses revealed no significant interactions between meal timing, meal skipping, or shiftwork and socioeconomic status or lifestyle regarding CircS. These findings highlight the importance of aligning mealtimes with circadian rhythms for improved circadian health.

## 1. Introduction

Endogenous clocks in the body govern circadian rhythms, which regulate sleep–wake cycles, cognitive function, and physiological processes such as heart rate, blood pressure, hormonal activity, and more [1]. The presence of intrinsic periodical cycles in living organisms, which occur in the absence of light or any other external stimuli, was first introduced in the beginning of the 18th century in plants [2]. These findings were expanded on throughout the centuries and provided key insights into the average cycle of rhythms occurring naturally every 24 h—hence termed “circadian”—and the role of external stimuli in synchronizing them. Light is the main regulator of the rhythms generated by the master biological clock in the suprachiasmatic nucleus of the hypothalamus [3]. The circadian clock follows a diurnal pattern, synchronizing human physiology according to time of day [4], and drives the rhythmicity of peripheral clocks throughout the body, including the liver, adipose tissue, intestine, skeletal muscles, and other organs [5]. While the master clock uses light as its main cue to express genes that regulate its function, peripheral clocks are primarily stimulated by food [6]. Traditionally, research has focused on the role of diet and nutrition in cardiometabolic disease risk, but mealtime is emerging as another risk factor. Timing of food intake appears to influence metabolism and chronic disease risk, and these metabolic disturbances, among other molecular mechanisms, may have an underlying circadian rhythm component [7].

Increasing evidence shows that circadian disruption may be responsible for type 2 diabetes mellitus (T2DM), cardiovascular disease (CVD), and hypertension in addition to commonly co-occurring conditions like sleep disturbances, depression, and metabolic dysfunction-associated steatotic liver disease (MASLD), previously known as non-alcoholic fatty liver disease [8]. Collectively, these conditions constitute ‘Circadian Syndrome’, as proposed by Zimmet and colleagues [8].

The body’s circadian clock impacts various cardiovascular functions, including endothelial function, blood pressure, thrombus formation, and heart rate, and the occurrence of cardiovascular events such as arrhythmias, stroke, and myocardial infarction follows a circadian rhythm [9]. Similarly, in the liver, the circadian clock regulates the accumulation of triglycerides, oxidative stress, mitochondrial dysfunction, and inflammation, and these contribute to the development of MASLD [10]. Mistimed food intake, for instance, during the biological night, when circadian rhythms related to digestion are operating differently from the day, may impair glucose tolerance and insulin function, a risk factor for T2DM [11]. Depressive disorders also appear to be linked with circadian disruption [12,13], and some of their treatments include light therapy, perhaps as a measure to realign circadian rhythms [14,15,16]. Similarly, sleep disturbances and short sleep are associated with circadian disruptions resulting from the misalignment of the sleep–wake, light–dark, and feeding–fasting cycles [17].

Studies have reported associations between CircS and unhealthy dietary patterns, stroke, kidney stones, overactive bladder, and testosterone deficiency [18,19,20,21,22]. CircS also appears to have a mediating effect in the relationship between air pollution and CVD [23]. Additionally, CircS is a more sensitive predictor for CVD and lower urinary tract symptoms than Metabolic Syndrome (MetS) [24,25]. 

Late meals and irregular intake, behaviors which are also common among shift workers, have been linked with circadian rhythm disturbances [26]. Currently, no study has assessed the associations of meal timing, meal skipping, shiftwork, and CircS. This study aims to use data from the National Health and Nutrition Examination Survey (NHANES) to examine the associations of CircS with meal timing, meal skipping, and shiftwork and to investigate any interaction with sociodemographic and lifestyle factors.

## 2. Materials and Methods

### 2.1. Study Design and Sample

The study sample included NHANES data from the surveys conducted between 2005 and 2016. The NHANES is conducted annually in the US to assess the health of the non-institutionalized population, using a probability sampling design for participant recruitment. Various assessment methods are used, including surveys and physical, medical, dental, and biochemical tests. 

**Figure 1:**
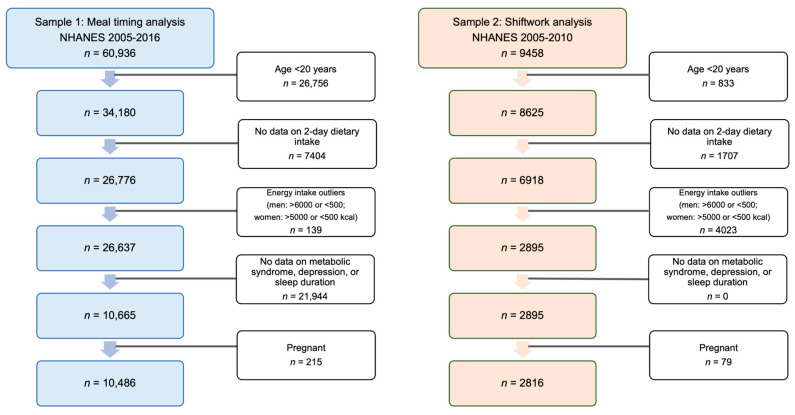
Sample flowchart for meal timing and shiftwork analysis. The white boxes represent the exclusion criteria and excluded subjects. Abbreviations: NHANES, National Health and Nutrition Examination Survey.

In this study, six NHANES cycles were combined to generate an analytical sample of 10,486 adults who were 20 years and older (Figure 1). Exclusion criteria included the following: (1) implausible energy intake based on two 24 h food recalls (defined as an intake of <500 kcal and >6000 kcal in men, and <500 kcal and >5000 kcal intake in women); (2) participants with incomplete data on CircS components; and (3) pregnant women, due to irregular patterns of sleep and hormonal and metabolic changes during pregnancy [27].

### 2.2. Outcome Variable

#### Circadian Syndrome

Trained staff conducted anthropometric and biochemical assessments in the homes of participants and in mobile examination centers. CircS was defined as the presence of 4 or more of the following components, as outlined by Shi and colleagues [24]: elevated waist circumference (≥88 cm in women and ≥102 cm in men), elevated fasting glucose (≥100 mg/dL or taking antihyperglycemic medication), elevated triglyceride (≥150 mg/dL serum triglyceride or taking medication), reduced HDL-cholesterol (serum HDL-C < 40 mg/dL in men and <50 mg/dL in women or taking medication), elevated blood pressure (≥130 mmHg systolic or a ≥85 mmHg diastolic blood pressure or taking antihypertensive medication), short sleep (self-reported <6 h per day), and depression symptoms (≥5 score on the Patient Health Questionnaire (PHQ-9)). A detailed description of CircS is published elsewhere [18]. 

### 2.3. Exposure Variables 

#### 2.3.1. Meal Timing and Meal Skipping

The timing of food intake was assessed using the first 24 h dietary recall interviews, which included the question “What time did you begin to eat/drink the meal/food?” In this study, the meal timing variable of “mid-eating time” was constructed as the midpoint of intake between breakfast and dinner, similar to prior studies; however, it differed in the categorization. While some studies used the midpoint of intake to classify participants as early or late eaters [28,29,30], our approach was different. The mid-eating time was dichotomized into a favorable mealtime (normal eaters, between 12:30 and 13:15) and unfavorable mealtime (abnormal eaters) using a data-driven approach. To identify the favorable midpoint mealtime, we categorized the midpoint mealtime as quartiles in a multivariable logistic regression model with CircS as the outcome variable and examined the odds ratios. Those who skipped meals (breakfast, lunch, or dinner) were categorized as meal skippers. 

#### 2.3.2. Shiftwork

Shiftwork was assessed using data from the 2005–2010 NHANES cycles, containing 9458 participants, as the cycles proceeding these did not include information on shiftwork. This was determined using the question “Which best describes hours worked?” Participants with any of the following responses were recoded as “yes” for shiftwork: (1) a regular evening shift; (2) a regular night shift; and (3) a rotating shift. The rest were recoded as “no” for shiftwork. Of the 9458 participants with information on shiftwork, 2816 were included in the analysis following the exclusion criteria described above (Figure 1).

### 2.4. Covariates 

Covariates included in the analysis were pre-selected based on current knowledge: age, sex, ethnicity (Non-Hispanic White, Non-Hispanic Black, Mexican American, and Other), energy intake measured via two 24 h recalls, physical activity (measured as Metabolic Equivalent of Task (MET) minutes per week using data from the validated Global Physical Activity Questionnaire and recoded as <600, 600–1200, and ≥1200 MET min/week), education (lower than 11th grade, high school, some college, and higher than college), smoking status (never, former, or current smoker), alcohol (categorized as “yes” or “no” through self-reported consumption over the previous 12 months), and Healthy Eating Index 2015 (HEI-2015). The HEI-2015 is a validated tool for examining how well an individual’s food intake aligns with the Dietary Guidelines for Americans [31]. It has 13 components, 9 of which highlight adequacy (foods to select more often for optimum health), and 4 components that focus on moderation (items to limit). The scores range from 0–100, and higher HEI-2015 scores reflect a better diet quality. The HEI-2015 scores were divided into quartiles in the analysis. The socioeconomic status (SES) was evaluated by dividing family income by the poverty threshold and classifying it into three categories: <1.3, 1.3–3.5, and >3.5 Poverty Income Ratio.

### 2.5. Statistical Analyses

Sample characteristics were presented according to the midpoint of intake or meal skipping if meals were omitted. To test the differences between groups, Chi-square test and ANOVA were used for categorical and continuous variables, respectively. Due to the skewed distribution and wide dispersion of values in each quartile of meal timing, the interquartile range (IQR) was used to define the time ranges in each corresponding quartile. Multivariable logistic regression was performed to examine the associations of CircS with meal timing, meal skipping, and shiftwork. Three multivariable models were used: model 1, adjusted for age, sex, ethnicity, and energy intake; model 2, further adjusted for physical activity, education level, smoking status, and alcohol consumption; and model 3, further adjusted for quartiles of the HEI. Sampling weights were used to account for the complex survey design of NHANES data. In subgroup analyses, potential effect modifications of CircS with meal timing and shiftwork by various factors (age, sex, ethnicity, energy intake, educational level, physical activity, smoking status, and alcohol consumption) were explored by adding product terms into the logistic regression models. All the analyses were performed using STATA 18 (Stata Corporation, College Station, TX, USA). Statistical significance was considered when *p*-values were less than 0.05.

## 3. Results

### 3.1. Sample Characteristics

In total, 10,486 adults (50.9% women) with a mean (SD) age of 50.3 (17.6) years were included in the analysis (Table 1). The unweighted prevalence of meal skipping was high (*n* = 4372, 41.7%). Meal skippers tended to consume fewer calories than others, and consumed less protein, fat, and carbohydrate overall. Their HEI scores were also lower. Meal skippers were younger and tended to be men, smokers, and of Non-Hispanic Black or Mexican American descent. They were more likely to have lower education levels and income, elevated glucose and triglycerides, reduced HDL-C, and were more likely to report depression symptoms, short sleep, have Circadian Syndrome. Those with a favorable mid-eating time were more likely to be women, college educated, high-income earners, and of Non-Hispanic White descent.

**Figure 2:**
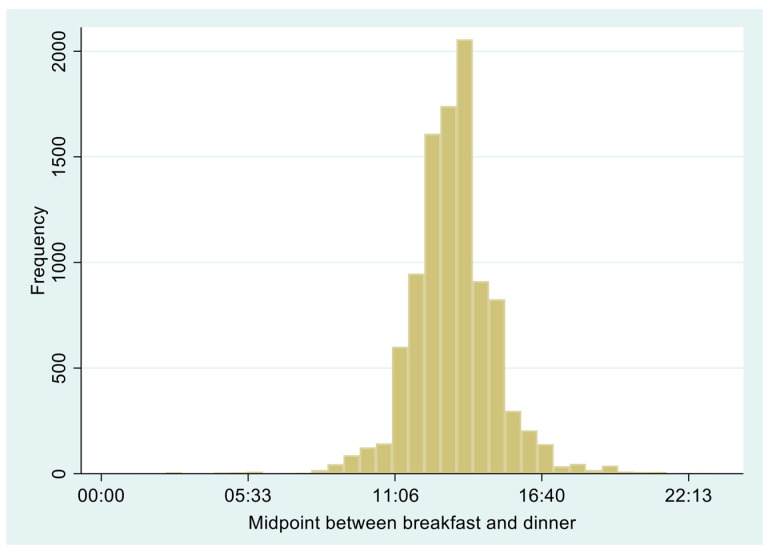
Distribution of midpoint between breakfast and dinner.

The median times for breakfast, lunch, and dinner were 8:20, 12:30, and 18:30, respectively. Figure 2 shows the distribution of the midpoint of intake between breakfast and dinner. The median (IQR) was 13:15 (12:30–14:15). Among the participants, the unweighted prevalence of shiftwork was 18.2%.

### 3.2. Meal Timing, Meal Skipping, and Circadian Syndrome 

Compared with a favorable mealtime (second quartile, midpoint of intake between 12:30 and 13:15), participants who skipped meals and those with an unfavorable mealtime had 39% (95%CI 1.16–1.67) and 24% (95%CI 1.07–1.44) higher odds of having CircS, respectively (Table 2). The positive association remained after adjusting for the HEI. 

The association of CircS by quartiles of the mid-eating time was also investigated (Table S1). There was a non-linear association between the midpoint of meal intake and CircS. Both early and late midpoints of meal intake were associated with an increased odds of having CircS. 

Among the components of CircS, meal skipping showed a positive association with all components except elevated triglycerides and blood pressure (Table 3). Those who skipped meals had ORs of 1.23 for central obesity (95%CI 1.03–1.47), 1.29 (95%CI 1.10–1.50) for elevated glucose, 1.22 (95%CI 1.04–1.44) for low HDL-C, 1.57 (95%CI 1.31–1.89) for depressive symptoms, and 1.37 for short sleep (95%CI 1.17–1.61).

Compared to favorable mealtimes, unfavorable mealtimes were associated with 20% higher odds of elevated glucose and short sleep, and 38% higher odds of depressive symptoms (Table 3). No associations were found between unfavorable mealtimes and other CircS components. Similar to meal skippers, participants with unfavorable mealtimes had the highest odds of depressive symptoms and short sleep among all the components of CircS.

### 3.3. Shiftwork and Circadian Syndrome 

Shiftwork was independently associated with CircS (Table 4). Compared to non-shift workers, a 40% higher likelihood of having CircS in shift workers was seen in model 2, and this significant association persisted in the fully adjusted model (OR = 1.37; 95%CI 1.01–1.87). The Healthy Eating Index did not significantly attenuate the relationship between shiftwork and CircS. 

### 3.4. Subgroup Analyses

Subgroup analyses revealed no significant interactions between mealtimes, SES, and lifestyle in relation to CircS (Table 5). However, the association of CircS with unfavorable mealtimes and meal skippers was only observed in women, middle-aged adults, individuals with lower education levels and HEI scores, non-smokers, and those of Non-Hispanic White descent. Additionally, meal skippers between the ages of 20 and 39, those within the lowest and highest categories of income and physical activity level, high school and college graduates, former smokers, and individuals with the highest HEI scores were positively associated with CircS.

Similarly, although the interaction of shiftwork, SES, and lifestyle in relation to CircS did not reach statistical significance, the association of shiftwork and CircS was seen among men but not women (Table 6).

## 4. Discussion

In this large, nationally representative study, we analyzed the associations of CircS—defined by expanding the MetS criteria to include short sleep and depression—with meal timing, meal skipping, and shiftwork. The prevalence of CircS, unfavorable mealtimes, and meal skipping was greater than 40% in this sample of US adults. An unfavorable mealtime, defined by the midpoint between breakfast and dinner time, skipping meals, and shiftwork were positively associated with CircS. The median (IQR) value of the midpoint was 13:15 (12:30–14:15). No effect modifications were observed between meal timing, meal skipping, shiftwork, and sociodemographic and lifestyle factors in relation to CircS.

### 4.1. Comparison with Other Studies 

Our findings on meal timing are in line with emerging evidence identifying the time of eating as a risk factor for impaired metabolic outcomes. Late eating has been associated with CircS components such as elevated glucose [32], waist circumference [30], and triglycerides [30] and low HDL-C [32] and sleep duration [33]. The effect of late eating on cardiometabolic risk factors and weight loss success after a weight loss intervention was investigated in a cross-sectional analysis [30]. Compared to early eaters, late eaters had elevated triglycerides, greater BMIs, and a reduced insulin sensitivity at baseline and were more likely to have weight loss barriers [30]. In an RCT, a late dinner induced nocturnal glucose impairment and reduced the oxidation and mobilization of fatty acids, especially in habitual early sleepers, compared to an early isocaloric dinner [34]. In a Japanese cohort, late eating was associated with obesity, dyslipidemia, and MetS, the latter only in women [35]. In our study, we created a reference midpoint of meal intake to categorize favorable or unfavorable mealtimes in relation to CircS. When the mid-eating time between breakfast and dinner was divided into quartiles, the association with CircS was significantly U-shaped. Both early and late eaters had an increased likelihood of having CircS and were categorized under unfavorable meal timing accordingly. This quartile also included those with a much earlier mid-eating time between breakfast and dinner, ranging from 1:00 a.m. to 12:30 p.m. It reflected individuals with mistimed sleeping and eating habits that continued throughout the night and were not aligned with circadian rhythms.

Similarly, meal skipping is also associated with MetS [36], all-cause and CVD mortality [37], and lower HEI scores [38]. The findings on breakfast versus dinner skipping are conflicting. In a cross-sectional analysis among Korean adults, women who skipped breakfast had higher odds of glucose and triglyceride metabolism impairment, while skipping dinner was associated with a favorable fasting glucose [36]. Another study found no association between skipping breakfast and MetS [39]. In the current study, meal skippers had a higher likelihood of CircS. Compared to other main meals, skipping breakfast showed the strongest association with CircS (results not shown). While other skippers were not significantly associated with CircS, dinner skippers tended to have lower odds of CircS, as corroborated by the existing literature.

Finally, the findings on shiftwork and CircS in the present study are consistent with the previous literature. Circadian disruptions manifesting as MetS and short sleep have been widely reported in shift workers [40]. 

### 4.2. Potential Mechanisms 

Over the past decade, Time-Restricted Eating (TRE)—limiting energy intake to a specific timeframe within a 24 h day—has been investigated as a potential approach to improve metabolic risk factors. Its effectiveness may depend on the length of the feeding window and the timing of food intake throughout the day. Confining meals to the morning, in line with the biological clock, as opposed to the evening has been associated with an improved insulin sensitivity, and lower inflammation, oxidative stress, and blood pressure [41,42,43,44]. A network meta-analysis of 12 RCTs on early versus later TRE revealed enhanced improvements in insulin resistance in early TRE (early vs. later TRE: −0.44; 95%CI −0.86 to −0.02; *p* < 0.05). While weight loss was similar in both interventions, early TRE was further associated with improved blood pressure and glucose metabolism [45]. 

As proposed by Zimmet et al. [8], metabolic alterations of glucose, blood pressure, and lipid profiles manifesting as cardiometabolic diseases are linked to disruptions in circadian rhythms. The internal circadian clock, consisting of central and peripheral clocks in the brain and peripheral tissues, respectively, generates these rhythms through transcription factors and gene expression, overseeing crucial physiological processes in the body, including metabolism [46]. The transcriptional pathways regulating the internal clock can be modulated and reset by a variety of signals, including light and feeding. For instance, breakfast consumption impacts the expression of clock genes and facilitates the typical patterns of rhythms [47]. In contrast, skipping breakfast impairs the expression of the internal clock and related genes and is associated with an elevated post-meal blood glucose response, irrespective of diabetes status [48]. The disruption of these pathways can lead to weight gain, an elevated blood pressure, impaired insulin sensitivity, and dyslipidemia. Modern lifestyle behaviors like shiftwork, prolonged exposure to artificial light, social jet lag (the misalignment between weekday and weekend sleep schedules), and nocturnal eating have been linked to circadian disruption [4].

### 4.3. Implications of Meal Timing and Skipping Meals

Consistent and timely meals are important due to the various health risks associated with irregular eating patterns and meal skipping. Adopting a one-meal-per-day approach may contribute to weight loss, without any benefit to lipid profiles [49], and may be associated with elevated fasting blood glucose levels [50]. Evidence suggests that skipping breakfast leads to an increased body weight and insulin resistance [51] and a reduction in the overall diet quality and intake of the recommended level of nutrients [52]. Notably, a higher prevalence of MetS was observed in those with inconsistent mealtimes in a Swedish cohort of 60-year-old adults [51]. Irregular meals are also associated with an increased likelihood of MetS later in life [53]. 

Furthermore, prior evidence indicates that meal timing may be related to diet quality. In a Serbian study, earlier breakfast and dinner consumption were associated with an increased consumption of fruits and vegetables and higher Dietary Quality Scores (based on European Union Science Hub recommendations on fruits and vegetables, fiber, sodium, saturated fat, and sugar) [54]. Similarly, in a cross-sectional study of Brazilian women, those with an early midpoint eating time had healthier eating habits as measured by Brazilian HEI scores [55]. Nutrient-rich and diverse dietary patterns are crucial for disease prevention, and these findings emphasize the equally important role of consistent and appropriate meal timing.

### 4.4. Strengths and Limitations

This study has several noteworthy strengths. It is the first study to investigate CircS with variables associated to meal timing and shiftwork. Secondly, the use of a robust sampling design improves the generalizability of the findings to the general non-institutionalized US population. Furthermore, we controlled for multiple lifestyle and sociodemographic factors due to the extensive dataset. In addition, subgroup analyses allowed us to investigate interactions of meal timing, meal skipping, and shiftwork with sociodemographic and lifestyle factors in relation to CircS.

This study also has certain limitations, such as the lack of repeated measurements of CircS. Moreover, a self-reported sleep duration was used in the study rather than objective measures. The threshold for identifying symptoms of depression using the PHQ-9 was established at 5 instead of 10, specifically capturing mild depression, but the PHQ-9 is easy and efficient to use. While appropriate statistical models were used to account for confounding, the possibility of residual confounding remains, such as environmental factors including air pollution, noise, and the use of artificial lighting. Additionally, the cross-sectional design of this study limits the ability for causal inferences.

## 5. Conclusions

Unfavorable midpoint eating time, meal skipping, and shiftwork were adversely associated with CircS, highlighting the importance of scheduled meals and aligning mealtimes with circadian rhythms in reducing the likelihood of circadian disruption. Future research could employ objectively measured data for sleep parameters, e.g., smart watches, or a validated questionnaire. Additionally, future studies could investigate the potential use of wearable devices to monitor circadian rhythms and validate the use of CircS as a practical tool for measuring circadian disruption in different populations. Longitudinal studies are warranted to determine the long-term effects of circadian rhythm disruptions on cardiometabolic outcomes and to identify the optimal timing of meals for circadian health in all populations.

## Figures and Tables

**Table 1:** Sample characteristics by mid-eating time (between breakfast and dinner) or meal skipping.

	Total	Favorable (12:30–13:15)	Unfavorable	Meal Skipping	*p*-Value
	*n* = 10,486	*n* = 1577	*n* = 4537	*n* = 4372	
Energy intake (kcal/day)	2023.8 (781.8)	2066.3 (740.9)	2083.1 (769.2)	1946.8 (802.5)	<0.001
Protein intake (g/day)	80.2 (34.0)	83.5 (33.0)	83.5 (33.5)	75.5 (34.3)	<0.001
Fat intake (g/day)	76.6 (36.3)	78.6 (35.6)	79.4 (36.0)	73.1 (36.4)	<0.001
Carbohydrate intake (g/day)	246.5 (100.5)	251.5 (94.0)	252.9 (99.5)	238.2 (103.1)	<0.001
Healthy Eating Index	51.7 (12.0))	54.7 (12.2)	53.2 (12.1)	49.2 (11.3)	<0.001
Age (years)	50.3 (17.6)	54.3 (17.2)	50.4 (17.2)	48.7 (17.9)	<0.001
Sex					<0.001
Men	5147 (49.1%)	724 (45.9%)	2128 (46.9%)	2295 (52.5%)	
Women	5339 (50.9%)	853 (54.1%)	2409 (53.1%)	2077 (47.5%)	
Ethnicity					<0.001
Non-Hispanic White	4973 (47.4%)	1002 (63.5%)	2304 (50.8%)	1667 (38.1%)	
Non-Hispanic Black	2002 (19.1%)	176 (11.2%)	745 (16.4%)	1081 (24.7%)	
Mexican American	1587 (15.1%)	180 (11.4%)	678 (14.9%)	729 (16.7%)	
Other	1924 (18.3%)	219 (13.9%)	810 (17.9%)	895 (20.5%)	
Education					<0.001
<11 grade	2494 (23.8%)	270 (17.1%)	898 (19.8%)	1326 (30.4%)	
High school	2400 (22.9%)	326 (20.7%)	964 (21.3%)	1110 (25.4%)	
Some college	3043 (29.0%)	467 (29.6%)	1353 (29.8%)	1223 (28.0%)	
Higher than college	2541 (24.3%)	513 (32.6%)	1318 (29.1%)	710 (16.3%)	
Smoking					<0.001
Never	5694 (54.3%)	884 (56.1%)	2572 (56.7%)	2238 (51.2%)	
Former	2698 (25.7%)	450 (28.5%)	1214 (26.8%)	1034 (23.7%)	
Current smoker	2090 (19.9%)	243 (15.4%)	749 (16.5%)	1098 (25.1%)	
Alcohol intake (past 12 months)					0.004
No	1938 (18.5%)	264 (16.7%)	797 (17.6%)	877 (20.1%)	
Yes	7143 (68.1%)	1078 (68.4%)	3141 (69.2%)	2924 (66.9%)	
Missing	1405 (13.4%)	235 (14.9%)	599 (13.2%)	571 (13.1%)	
BMI (kg/m^2^)	29.1 (6.7)	28.9 (6.6)	28.9 (6.7)	29.3 (6.8)	0.012
Leisure time physical activity (MET min/week)					<0.001
<600	4153 (39.6%)	620 (39.3%)	1711 (37.7%)	1822 (41.7%)	
600–1200	1218 (11.6%)	211 (13.4%)	557 (12.3%)	450 (10.3%)	
≥1200	5114 (48.8%)	746 (47.3%)	2268 (50.0%)	2100 (48.0%)	
Ratio of family income to poverty					<0.001
<1.30	2904 (29.9%)	309 (21.1%)	1073 (25.4%)	1522 (38.0%)	
1.3–3.5	3717 (38.3%)	538 (36.7%)	1585 (37.5%)	1594 (39.8%)	
>3.5	3084 (31.8%)	619 (42.2%)	1571 (37.1%)	894 (22.3%)	
Hypertension	3871 (37.0%)	653 (41.4%)	1657 (36.6%)	1561 (35.8%)	<0.001
Central obesity	6056 (57.8%)	937 (59.4%)	2599 (57.3%)	2520 (57.6%)	0.33
Elevated glucose	5567 (53.1%)	824 (52.3%)	2377 (52.4%)	2366 (54.1%)	0.20
Elevated triglycerides	4495 (42.9%)	676 (42.9%)	1905 (42.0%)	1914 (43.8%)	0.23
Reduced HDL-C	4724 (45.1%)	702 (44.5%)	2024 (44.6%)	1998 (45.7%)	0.53
Elevated blood pressure	5137 (49.0%)	831 (52.7%)	2196 (48.4%)	2110 (48.3%)	0.006
Depression symptoms	2421 (23.1%)	288 (18.3%)	1006 (22.2%)	1127 (25.8%)	<0.001
Short sleep	3657 (34.9%)	443 (28.1%)	1531 (33.7%)	1683 (38.5%)	<0.001
Metabolic Syndrome	5124 (48.9%)	796 (50.5%)	2190 (48.3%)	2138 (48.9%)	0.32
Circadian Syndrome	4331 (41.3%)	632 (40.1%)	1828 (40.3%)	1871 (42.8%)	0.032

Data are presented as mean (SD) for continuous measures and *n* (%) for categorical measures. Meal skipping was defined as skipping breakfast, lunch, or dinner based on first 24 h dietary recall. *p*-values reflect the overall difference among the groups.

**Table 2:** Odds ratio (95%CI) for Circadian Syndrome by mid-eating time among adults participating in NHANES 2005–2016 (*n* = 10,486).

	Unadjusted		Model 1		Model 2		Model 3	
	Coef. (95% CI)	*p*-Value	Coef. (95% CI)	*p*-Value	Coef. (95% CI)	*p*-Value	Coef. (95% CI)	*p*-Value
Favorable mealtime (12:30–13:15)								
Favorable	1.00		1.00		1.00		1.00	
Unfavorable	1.08 (0.94–1.25)	0.270	1.28 (1.11–1.48)	0.001	1.26 (1.08–1.46)	0.003	1.24 (1.07–1.44)	0.005
Meal skipping	1.25 (1.06–1.48)	0.010	1.70 (1.43–2.02)	<0.001	1.47 (1.22–1.76)	<0.001	1.39 (1.16–1.67)	<0.001

Model 1 adjusted for age, sex, ethnicity, and energy intake. Model 2 further adjusted for physical activity, education, smoking, and alcohol intake. Model 3 further adjusted for the Healthy Eating Index (quartiles).

**Table 3:** Odds ratios (95%CI) for components of CircS by quartiles of eating time among adults participating in NHANES 2005–2016 (*n* = 10,486).

	Favorable Mealtime ^a^	Unfavorable Mealtime		Meal Skipping ^b^	
		OR (95%CI)	*p*-Value	OR (95%CI)	*p*-Value
Central obesity	1	1.08 (0.93–1.27)	0.313	1.23 (1.03–1.47)	0.026
Elevated glucose	1	1.20 (1.03–1.40)	0.019	1.29 (1.10–1.50)	0.002
Elevated triglycerides	1	1.08 (0.91–1.29)	0.385	1.19 (1.00–1.43)	0.051
Low HDL-C	1	1.13 (0.98–1.30)	0.083	1.22 (1.04–1.44)	0.015
Elevated blood pressure	1	1.04 (0.85–1.26)	0.71	1.01 (0.83–1.22)	0.937
Depressive symptom	1	1.38 (1.14–1.68)	0.001	1.57 (1.31–1.89)	<0.001
Short sleep	1	1.20 (1.02–1.42)	0.031	1.37 (1.17–1.61)	<0.001

^a^ Favorable mealtime was defined as a midpoint of intake between 12:30 and 13:15. It was the second quartile of the midpoint of meal intake in the sample. All mealtimes with a midpoint outside this range were defined as “unfavorable mealtime”. ^b^ Meal skipping was defined as skipping breakfast, lunch, or dinner based on the first 24 h dietary recall. Models adjusted for age, gender, ethnicity, energy intake, leisure time physical activity, education, smoking, and alcohol intake.

**Table 4:** Odds ratio (95%CI) for Circadian Syndrome by shiftwork status (*n* = 2816).

Shiftwork			
	No	Yes	*p*-Value
Unadjusted	1.00	1.23 (0.89–1.70)	0.198
Model 1	1.00	1.53 (1.12–2.10)	0.009
Model 2	1.00	1.40 (1.02–1.93)	0.038
Model 3	1.00	1.37 (1.01–1.87)	0.044

Values are odds ratios (95%CI) from logistic regression. Model 1 adjusted for age, gender, ethnicity, and energy intake. Model 2 further adjusted for physical activity, education, smoking, and alcohol intake. Model 3 further adjusted for the Healthy Eating Index (quartiles).

**Table 5:** Subgroup analyses of the association between the midpoint of mealtime and CircS among adults participating in NHANES 2005–2016 (*n* = 10,486).

	Favorable Mealtime ^a^ (12:30−13:15)				
	Favorable	Unfavorable	Meal Skipping ^b^	*p* for Trend ^c^	*p* for Interaction
Ethnicity					0.095
Non-Hispanic White	1.00	1.33 (1.12−1.58)	1.61 (1.27−2.03)	<0.001	
Non-Hispanic Black	1.00	0.80 (0.49−1.30)	0.84 (0.54−1.31)	0.665	
Mexican American	1.00	1.05 (0.64−1.73)	1.07 (0.71−1.59)	0.748	
Others	1.00	0.99 (0.60−1.65)	1.18 (0.71−1.97)	0.353	
Sex					0.192
Men	1.00	1.09 (0.85−1.40)	1.41 (1.07−1.84)	0.005	
Women	1.00	1.43 (1.17−1.74)	1.51 (1.19−1.92)	0.002	
Age					0.549
20−39	1.00	1.08 (0.73−1.59)	1.49 (1.01−2.20)	0.010	
40−59	1.00	1.41 (1.08−1.85)	1.68 (1.24−2.27)	0.001	
60+	1.00	1.14 (0.90−1.43)	1.23 (0.93−1.63)	0.150	
Ratio of family income to poverty					0.933
<1.30	1.00	1.47 (0.97−2.25)	1.58 (1.11−2.27)	0.011	
1.3−3.5	1.00	1.20 (0.90−1.62)	1.36 (0.98−1.87)	0.062	
>3.5	1.00	1.30 (1.00−1.70)	1.57 (1.13−2.20)	0.008	
Education					0.259
<11 grade	1.00	1.60 (1.11−2.32)	1.62 (1.13−2.33)	0.033	
High school	1.00	1.36 (0.96−1.92)	1.68 (1.17−2.42)	0.005	
Some college	1.00	1.30 (0.99−1.72)	1.31 (0.94−1.84)	0.171	
>college	1.00	1.09 (0.83−1.44)	1.50 (1.08−2.08)	0.017	
Leisure time physical activity (MET min/week)					0.126
<600	1.00	1.24 (0.93−1.66)	1.41 (1.02−1.93)	0.031	
600−1200	1.00	1.78 (1.03−3.07)	1.49 (0.85−2.62)	0.227	
≥1200	1.00	1.18 (0.93−1.50)	1.50 (1.17−1.92)	<0.001	
Smoking					0.659
Never	1.00	1.27 (1.01−1.62)	1.55 (1.17−2.04)	0.002	
Former	1.00	1.16 (0.86−1.57)	1.39 (1.01−1.90)	0.036	
Current smoker	1.00	1.40 (0.95−2.07)	1.45 (0.97−2.16)	0.124	
Healthy Eating Index					0.374
Q1	1.00	1.54 (1.08−2.20)	1.68 (1.15−2.47)	0.022	
Q2	1.00	1.08 (0.77−1.53)	1.27 (0.87−1.87)	0.182	
Q3	1.00	1.11 (0.80−1.53)	1.22 (0.87−1.69)	0.229	
Q4	1.00	1.37 (1.00−1.89)	1.67 (1.16−2.41)	0.007	

^a^ Favorable mealtime was defined as the midpoint of meal intake between 12:30 and 13:15. It was the second quartile of the midpoint of meal intake in the sample. All mealtimes with a midpoint outside this range were defined as “unfavorable mealtime”. ^b^ Meal skipping was defined as skipping breakfast, lunch, or dinner based on the first 24 h dietary recall. ^c^ p-trend was tested using ordinal numbers 1, 2, and 3 for favorable mealtime, unfavorable mealtime, and meal skipping in the multivariable logistic model adjusted for age, gender, ethnicity, energy intake, leisure time physical activity, education, smoking, and alcohol intake.

**Table 6:** Subgroup analyses of the association between shiftwork and Circadian Syndrome among adults participating in NHANES 2005-2010 (*n* = 2816).

Shiftwork				
	No	Yes	*p* for Trend	*p* for Interaction
Ethnicity				0.510
Non-Hispanic White	1.00	1.38 (0.89–2.14)	0.141	
Non-Hispanic Black	1.00	1.20 (0.69–2.09)	0.509	
Mexican American	1.00	1.05 (0.50–2.18)	0.899	
Others	1.00	1.95 (1.01–3.76)	0.047	
Sex				0.065
Men	1.00	1.86 (1.22–2.84)	0.005	
Women	1.00	0.87 (0.49–1.55)	0.636	
Age group				0.912
20–39	1.00	1.47 (0.92–2.35)	0.109	
40–59	1.00	1.41 (0.89–2.22)	0.139	
60+	1.00	1.35 (0.55–3.34)	0.507	
Ratio of family income to poverty				0.686
<1.30	1.00	1.51 (0.72–3.18)	0.269	
1.3–3.5	1.00	1.30 (0.84–2.01)	0.228	
>3.5	1.00	1.65 (1.06–2.57)	0.026	
Education				0.814
<11 grade	1.00	1.43 (0.65–3.14)	0.370	
High school	1.00	1.15 (0.58–2.26)	0.688	
Some college	1.00	1.35 (0.89–2.05)	0.153	
Higher than college	1.00	1.78 (0.97–3.27)	0.063	
Leisure time physical activity (MET min/week)				0.300
<600	1.00	1.12 (0.61–2.06)	0.702	
600–1200	1.00	1.69 (0.81–3.55)	0.161	
≥1200	1.00	1.50 (1.07–2.12)	0.021	
Smoking				0.485
Never	1.00	1.42 (0.97–2.08)	0.073	
Former	1.00	1.05 (0.63–1.75)	0.846	
Current smoker	1.00	1.70 (0.94–3.09)	0.081	

Regression models adjusted for age, gender, ethnicity, energy intake, leisure time physical activity, education, smoking, and alcohol intake. Stratification variables were not adjusted in the corresponding models.

## Data Availability

The data used in the study are publicly available on the NHANES website.

## Supplementary Materials

The following supporting information can be downloaded at: https://www.mdpi.com/article/10.3390/nu16111581/s1, Table S1: Odds ratio (95% CI) for Circadian Syndrome by quartiles of mid-eating time between breakfast and dinner among adults participating in NHANES 2005–2016 (*n* = 10,486).

## References

[1] Allada R., Bass J. (2021). Circadian Mechanisms in Medicine. N. Engl. J. Med..

[2] The Nobel Prize in Physiology or Medicine 2017. https://www.nobelprize.org/prizes/medicine/2017/advanced-information/.

[3] Berson D.M., Dunn F.A., Takao M. (2002). Phototransduction by Retinal Ganglion Cells That Set the Circadian Clock. Science.

[4] Potter G.D.M., Skene D.J., Arendt J., Cade J.E., Grant P.J., Hardie L.J. (2016). Circadian Rhythm and Sleep Disruption: Causes, Metabolic Consequences, and Countermeasures. Endocr. Rev..

[5] Dibner C., Schibler U., Albrecht U. (2010). The Mammalian Circadian Timing System: Organization and Coordination of Central and Peripheral Clocks. Annu. Rev. Physiol..

[6] Pickel L., Sung H.-K. (2020). Feeding Rhythms and the Circadian Regulation of Metabolism. Front. Nutr..

[7] Panda S. (2016). Circadian Physiology of Metabolism. Science.

[8] Zimmet P., Alberti K.G.M.M., Stern N., Bilu C., El-Osta A., Einat H., Kronfeld-Schor N. (2019). The Circadian Syndrome: Is the Metabolic Syndrome and Much More!. J. Intern. Med..

[9] Crnko S., Du Pré B.C., Sluijter J.P.G., Van Laake L.W. (2019). Circadian Rhythms and the Molecular Clock in Cardiovascular Biology and Disease. Nat. Rev. Cardiol..

[10] Reinke H., Asher G. (2016). Circadian Clock Control of Liver Metabolic Functions. Gastroenterology.

[11] Mason I.C., Qian J., Adler G.K., Scheer F.A.J.L. (2020). Impact of Circadian Disruption on Glucose Metabolism: Implications for Type 2 Diabetes. Diabetologia.

[12] Kronfeld-Schor N., Einat H. (2012). Circadian Rhythms and Depression: Human Psychopathology and Animal Models. Neuropharmacology.

[13] Logan R.W., McClung C.A. (2019). Rhythms of Life: Circadian Disruption and Brain Disorders across the Lifespan. Nat. Rev. Neurosci..

[14] Crouse J.J., Carpenter J.S., Song Y.J.C., Hockey S.J., Naismith S.L., Grunstein R.R., Scott E.M., Merikangas K.R., Scott J., Hickie I.B. (2021). Circadian Rhythm Sleep-Wake Disturbances and Depression in Young People: Implications for Prevention and Early Intervention. Lancet Psychiatry.

[15] Even C., Schröder C.M., Friedman S., Rouillon F. (2008). Efficacy of Light Therapy in Nonseasonal Depression: A Systematic Review. J. Affect. Disord..

[16] Chang C.-H., Liu C.-Y., Chen S.-J., Tsai H.-C. (2018). Efficacy of Light Therapy on Nonseasonal Depression among Elderly Adults: A Systematic Review and Meta-Analysis. Neuropsychiatr. Dis. Treat..

[17] Almoosawi S., Vingeliene S., Gachon F., Voortman T., Palla L., Johnston J.D., Van Dam R.M., Darimont C., Karagounis L.G. (2019). Chronotype: Implications for Epidemiologic Studies on Chrono-Nutrition and Cardiometabolic Health. Adv. Nutr..

[18] Akbar Z., Shi Z. (2023). Dietary Patterns and Circadian Syndrome among Adults Attending NHANES 2005–2016. Nutrients.

[19] Wang Y., Yang L., Zhang Y., Liu J. (2022). Relationship between Circadian Syndrome and Stroke: A Cross-Sectional Study of the National Health and Nutrition Examination Survey. Front. Neurol..

[20] Xiao Y., Yin S., Bai Y., Yang Z., Wang J., Cui J., Wang J. (2023). Association between Circadian Syndrome and the Prevalence of Kidney Stones in Overweight Adults: A Cross-Sectional Analysis of NHANES 2007–2018. BMC Public Health.

[21] Xiao Y., Yin S., Wang J., Cui J., Yang Z., Wang J., Bai Y. (2023). A Positive Association between the Prevalence of Circadian Syndrome and Overactive Bladder in United States Adults. Front. Public Health.

[22] Xiao Y., Yin S., Cui J., Bai Y., Yang Z., Wang J., Wang J. (2023). Association between the Prevalence Rates of Circadian Syndrome and Testosterone Deficiency in US Males: Data from NHANES (2011–2016). Front. Nutr..

[23] Hu X., Nie Z., Ou Y., Lin L., Qian Z., Vaughn M.G., McMillin S.E., Zhou Y., Wu Y., Dong G. (2023). Long-Term Exposure to Ambient Air Pollution, Circadian Syndrome and Cardiovascular Disease: A Nationwide Study in China. Sci. Total Environ..

[24] Shi Z., Tuomilehto J., Kronfeld-Schor N., Alberti G.K., Stern N., El-Osta A., Bilu C., Einat H., Zimmet P. (2021). The Circadian Syndrome Predicts Cardiovascular Disease Better than Metabolic Syndrome in Chinese Adults. J. Intern. Med..

[25] Xiong Y., Zhang F., Wu C., Zhang Y., Huang X., Qin F., Yuan J. (2021). The Circadian Syndrome Predicts Lower Urinary Tract Symptoms Suggestive of Benign Prostatic Hyperplasia Better Than Metabolic Syndrome in Aging Males: A 4-Year Follow-Up Study. Front. Med..

[26] Meléndez-Fernández O.H., Liu J.A., Nelson R.J. (2023). Circadian Rhythms Disrupted by Light at Night and Mistimed Food Intake Alter Hormonal Rhythms and Metabolism. Int. J. Mol. Sci..

[27] Lee K.A. (1998). Alterations in Sleep during Pregnancy and Postpartum: A Review of 30 Years of Research. Sleep Med. Rev..

[28] Lopez-Minguez J., Dashti H.S., Madrid-Valero J.J., Madrid J.A., Saxena R., Scheer F.A.J.L., Ordoñana J.R., Garaulet M. (2019). Heritability of the Timing of Food Intake. Clin. Nutr..

[29] Billingsley H.E., Canada J.M., Dixon D.L., Kirkman D.L., Bohmke N., Rotelli B., Kadariya D., Markley R., Van Tassell B.W., Celi F.S. (2022). Midpoint of Energy Intake, Non-Fasting Time and Cardiorespiratory Fitness in Heart Failure with Preserved Ejection Fraction and Obesity. Int. J. Cardiol..

[30] Dashti H.S., Gómez-Abellán P., Qian J., Esteban A., Morales E., Scheer F.A.J.L., Garaulet M. (2020). Late Eating Is Associated with Cardiometabolic Risk Traits, Obesogenic Behaviors, and Impaired Weight Loss. Am. J. Clin. Nutr..

[31] Kennedy E.T., Ohls J., Carlson S., Fleming K. (1995). The Healthy Eating Index: Design and Applications. J. Am. Diet. Assoc..

[32] Ali M., Reutrakul S., Petersen G., Knutson K.L. (2023). Associations between Timing and Duration of Eating and Glucose Metabolism: A Nationally Representative Study in the U.S. Nutrients.

[33] Iao S.I., Jansen E., Shedden K., O’Brien L.M., Chervin R.D., Knutson K.L., Dunietz G.L. (2021). Associations between Bedtime Eating or Drinking, Sleep Duration and Wake after Sleep Onset: Findings from the American Time Use Survey. Br. J. Nutr..

[34] Gu C., Brereton N., Schweitzer A., Cotter M., Duan D., Børsheim E., Wolfe R.R., Pham L.V., Polotsky V.Y., Jun J.C. (2020). Metabolic Effects of Late Dinner in Healthy Volunteers-A Randomized Crossover Clinical Trial. J. Clin. Endocrinol. Metab..

[35] Yoshida J., Eguchi E., Nagaoka K., Ito T., Ogino K. (2018). Association of Night Eating Habits with Metabolic Syndrome and Its Components: A Longitudinal Study. BMC Public Health.

[36] Park H., Shin D., Lee K.W. (2023). Association of Main Meal Frequency and Skipping with Metabolic Syndrome in Korean Adults: A Cross-Sectional Study. Nutr. J..

[37] Sun Y., Rong S., Liu B., Du Y., Wu Y., Chen L., Xiao Q., Snetselaar L., Wallace R., Bao W. (2023). Meal Skipping and Shorter Meal Intervals Are Associated with Increased Risk of All-Cause and Cardiovascular Disease Mortality among US Adults. J. Acad. Nutr. Diet..

[38] Zeballos E., Todd J.E. (2020). The Effects of Skipping a Meal on Daily Energy Intake and Diet Quality. Public Health Nutr..

[39] Jung J., Kim A.-S., Ko H.-J., Choi H.-I., Hong H.-E. (2020). Association between Breakfast Skipping and the Metabolic Syndrome: The Korea National Health and Nutrition Examination Survey, 2017. Medicina.

[40] Mohd Azmi N.A.S., Juliana N., Mohd Fahmi Teng N.I., Azmani S., Das S., Effendy N. (2020). Consequences of Circadian Disruption in Shift Workers on Chrononutrition and Their Psychosocial Well-Being. Int. J. Environ. Res. Public Health.

[41] Sutton E.F., Beyl R., Early K.S., Cefalu W.T., Ravussin E., Peterson C.M. (2018). Early Time-Restricted Feeding Improves Insulin Sensitivity, Blood Pressure, and Oxidative Stress Even without Weight Loss in Men with Prediabetes. Cell Metab..

[42] Jones R., Pabla P., Mallinson J., Nixon A., Taylor T., Bennett A., Tsintzas K. (2020). Two Weeks of Early Time-Restricted Feeding (ETRF) Improves Skeletal Muscle Insulin and Anabolic Sensitivity in Healthy Men. Am. J. Clin. Nutr..

[43] Ravussin E., Beyl R.A., Poggiogalle E., Hsia D.S., Peterson C.M. (2019). Early Time-Restricted Feeding Reduces Appetite and Increases Fat Oxidation but Does Not Affect Energy Expenditure in Humans. Obesity.

[44] Jamshed H., Beyl R.A., Della Manna D.L., Yang E.S., Ravussin E., Peterson C.M. (2019). Early Time-Restricted Feeding Improves 24-Hour Glucose Levels and Affects Markers of the Circadian Clock, Aging, and Autophagy in Humans. Nutrients.

[45] Liu J., Yi P., Liu F. (2023). The Effect of Early Time-Restricted Eating vs. Later Time-Restricted Eating on Weight Loss and Metabolic Health: A Network Meta-Analysis of Randomized Controlled Trials. J. Clin. Endocrinol. Metab..

[46] Bellet M.M., Orozco-Solis R., Sahar S., Eckel-Mahan K., Sassone-Corsi P. (2011). The Time of Metabolism: NAD+, SIRT1, and the Circadian Clock. Cold Spring Harb. Symp. Quant. Biol..

[47] BaHammam A.S., Pirzada A. (2023). Timing Matters: The Interplay between Early Mealtime, Circadian Rhythms, Gene Expression, Circadian Hormones, and Metabolism—A Narrative Review. Clocks Sleep.

[48] Jakubowicz D., Wainstein J., Landau Z., Raz I., Ahren B., Chapnik N., Ganz T., Menaged M., Barnea M., Bar-Dayan Y. (2017). Influences of Breakfast on Clock Gene Expression and Postprandial Glycemia in Healthy Individuals and Individuals with Diabetes: A Randomized Clinical Trial. Diabetes Care.

[49] Carlson O., Martin B., Stote K.S., Golden E., Maudsley S., Najjar S.S., Ferrucci L., Ingram D.K., Longo D.L., Rumpler W.V. (2007). Impact of Reduced Meal Frequency without Caloric Restriction on Glucose Regulation in Healthy, Normal-Weight Middle-Aged Men and Women. Metabolism.

[50] Laguzzi F., Salleber S., Gigante B., De Faire U., Hellenius M.L., Leander K. (2019). Irregular Eating Behavior and Incidence of Cardiovascular Disease: Results from a Swedish 60-Year-Old Cohort of Men and Women. Eur. Heart J..

[51] Sierra-Johnson J., Undén A.-L., Linestrand M., Rosell M., Sjogren P., Kolak M., De Faire U., Fisher R.M., Hellénius M.-L. (2008). Eating Meals Irregularly: A Novel Environmental Risk Factor for the Metabolic Syndrome. Obesity.

[52] Fanelli S., Walls C., Taylor C. (2021). Skipping Breakfast Is Associated with Nutrient Gaps and Poorer Diet Quality among Adults in the United States. Proc. Nutr. Soc..

[53] Wennberg M., Gustafsson P.E., Wennberg P., Hammarström A. (2016). Irregular Eating of Meals in Adolescence and the Metabolic Syndrome in Adulthood: Results from a 27-Year Prospective Cohort. Public Health Nutr..

[54] Djuric Z., Nikolic M., Zekovic M., Plegue M., Glibetic M. (2020). Association of Meal Timing with Dietary Quality in a Serbian Population Sample. BMC Nutr..

[55] Lima M.T.M., Nunes F.S.M., Custódio I.D.D., Carvalho K.P., Canto P.P.L., Paiva C.E., Crispim C.A., Paiva Maia Y.C. (2022). Eating Earlier and More Frequently Is Associated with Better Diet Quality in Female Brazilian Breast Cancer Survivors Using Tamoxifen. J. Acad. Nutr. Diet..

