# Supplementary material for: Unfavorable Mealtime, Meal Skipping, and Shiftwork Are Associated with Circadian Syndrome in Adults Participating in NHANES 2005–2016
Source: Nutrients. 2024 May 23;16(11):1581. doi: 10.3390/nu16111581 (PMC11173982; doi:10.3390/nu16111581)
Supplement: Supplementary file 1 [file nutrients-16-01581-s001.zip › nutrients-3005091-supplementary.pdf]

Unfavorable mealtime, meal skipping, and shift work are associated with circadian syndrome in adults participating in NHANES 2005-2016.

Zoha Akbar, Zumin Shi

Supplementary Table S1. Odds ratio (95%CI) for Circadian Syndrome by quartiles of mid-eating time between breakfast and dinner among adults attending NHANES 2005-2016 (*n* = 10,486).

	**Quartiles of Midpoint of Meal Intake**					
	**Q1**	**Q2**	**Q3**	**Q4**	***p*-Linear Trend**	***p* for Non-Linear Trend**
Unadjusted	1.43 (1.16-1.76)	1.00	1.01 (0.86-1.19)	0.79 (0.65-0.96)	<0.001	0.024
Model 1	1.31 (1.05-1.64)	1.00	1.25 (1.04-1.49)	1.23 (1.00-1.51)	0.935	0.015
Model 2	1.25 (0.99-1.58)	1.00	1.27 (1.05-1.52)	1.22 (0.98-1.51)	0.726	0.018
Model 3	1.24 (0.98-1.56)	1.00	1.25 (1.04-1.50)	1.20 (0.97-1.48)	0.787	0.022

The time range reflected in the quartiles is 1:00-12:30 in Q1; 12:30-13:15 in Q2; 13:20-14:15 in Q3; 14:22-23:00 in Q4.
P for non-linear association was tested using the cubic term of the quartiles of midpoint of meal intake using p.contrast command after logistic regression in Stata. Meal skippers were excluded in the analyses.
Model 1 adjusted for age, gender, race, energy intake.
Model 2 further adjusted for physical activity, education, smoking, and alcohol drinking.
Model 3 further adjusted for Healthy Eating Index (quartiles).
